# Single-carbon discrimination by selected peptides for individual detection of volatile organic compounds

**DOI:** 10.1038/srep09196

**Published:** 2015-03-17

**Authors:** Soomi Ju, Ki-Young Lee, Sun-Joon Min, Yong Kyoung Yoo, Kyo Seon Hwang, Sang Kyung Kim, Hyunjung Yi

**Affiliations:** 1 Center for BioMicrosystems, Brain Science Institute, Korea Institute of Science and Technology, Seoul. 136-791, Republic of Korea; 2 Center for Spintronics, Post-Silicon Semiconductor Institute, Korea Institute of Science and Technology, Seoul. 136-791, Republic of Korea; 3 Center for Neuro-medicine, Brain Science Institute, Korea Institute of Science and Technology, Seoul. 136-791, Republic of Korea

## Abstract

Although volatile organic compounds (VOCs) are becoming increasingly recognized as harmful agents and potential biomarkers, selective detection of the organic targets remains a tremendous challenge. Among the materials being investigated for target recognition, peptides are attractive candidates because of their chemical robustness, divergence, and their homology to natural olfactory receptors. Using a combinatorial peptide library and either a graphitic surface or phenyl-terminated self-assembled monolayer as relevant target surfaces, we successfully selected three interesting peptides that differentiate a single carbon deviation among benzene and its analogues. The heterogeneity of the designed target surfaces provided peptides with varying affinity toward targeted molecules and generated a set of selective peptides that complemented each other. Microcantilever sensors conjugated with each peptide quantitated benzene, toluene and xylene to sub-ppm levels in real time. The selection of specific receptors for a group of volatile molecules will provide a strong foundation for general approach to individually monitoring VOCs.

Over the last few years there has been a rapidly increasing interest in volatile organic compounds (VOCs). Several VOCs are known to be very harmful, with long-term effects on health. For example, inhaled benzene can cause cancers, mostly leukemia and other blood-related cancers^1^,^2^. In addition, several studies have recently reported that exhaled breath contains dozens of different chemicals that signify health conditions related to metabolic or infectious diseases^3^,^4^. Importantly, the majority of the exhaled chemicals are VOCs in the range of 30–200 Daltons according to gas chromatography-mass spectroscopy (GC-MS) analysis^5^,^6^. Therefore, accurate and facile analysis of VOCs might not only protect the living environment from frequently used hazardous gases, but might also provide a non-invasive means of monitoring health.

Alternative and simpler sensors to replace gas chromatography for facile analysis of gases have been intensively researched. Significant advances in nanomaterials and nanofabrications have led to the development of a variety of sensitive transducers^7^,^8^,^9^; however, the selectivity of sensors to detect individual VOCs in mixed samples remains a crucial challenge. Therefore, there is a significant focus on the enhancement of selectivity. Traditional semiconducting sensors have adopted various metal catalysts to differentiate organic molecules^10^. For example, a SnO_2_/V_2_O_5_ composite material was applied to discriminate benzene derivatives from other interfering VOCs. Using the catalytic reaction with V_2_O_5_ at 270°C, the signal from benzene was two times stronger than the signal from acetone and showed less than 20% difference from analogous gases such as toluene or xylene^11^. Pattern recognition of chemical reactions that change the chromatic character of metal-ligand complexes has also been used to identify VOCs. Tens of different dyes were arrayed to express patterns of irreversible color change caused by oxidized molecules. Using disposable arrays, 20 kinds of environmental VOCs could be recognized to the level of hundreds of ppb^12^. Explosives were also identifiable from their vapors using surface-enhanced Raman spectroscopy (SERS)^13^,^14^ and photo-thermal spectroscopy^15^ by tracing intrinsic NO_2_ peaks. Slightly volatile explosives were accumulated on the sensing surface and measured to the level of ppt upon irradiation. However, volatile gases such as pyridine were not detectable in a practical concentration range, and the methods are not applicable to common VOCs with high vapor pressure such as benzene and toluene.

Various recognition layers such as synthetic host molecules^16^, aromatic receptor molecules^17^, DNA^18^, and protein receptors have recently been reported to show improved selectivity toward volatile chemicals. For example, individual ‘selectors'^19^, small molecules selected from a synthesized chemical library, provide selectivity to individual VOCs. The most promising ‘selector' showed selectivity higher than 10:1 for cyclohexanone against tetrahydrofuran (THF) at a concentration level of 100 ppm. DNA assembled onto carbon nanotubes (CNT) could discriminate limonene enantiomers by opposite polarity of signal in the concentration range of several ppm^18^. Other biological recognition layers were also applied to realize unmet selectivity. Olfactory receptors have been developed by evolution toward volatile odorants. A human olfactory receptor settled on CNT could differentiate a series of butylates dissolved in water with extreme selectivity of 10^9^:1^20^. These findings suggest the potential of natural receptors in the selective detection of specific VOCs.

In this study, we demonstrate that peptides are promising receptors for the specific detection of VOCs with unprecedented discrimination. Peptide fragments of proteins are robust and chemically flexible, and more divergent than oligonucleotides^21^. We chose benzene as an important target VOC because it is a carcinogenic hazard and a potential biomarker for lung cancer. Moreover, no sensor has been reported that can individually distinguish benzene from its derivatives and the interfering VOCs that are abundant in breath. By screening a special peptide library designed for small molecules against a graphitic surface and a phenyl-functionalized self-assembled monolayer (SAM) we identified three interesting peptide sequences. We show that these screened peptides distinguish single-carbon deviations among benzene, toluene, and xylene (BTX) with exceptional selectivity and sensitivity.

## Results

### Screening of peptide receptors

**Figure 1:**
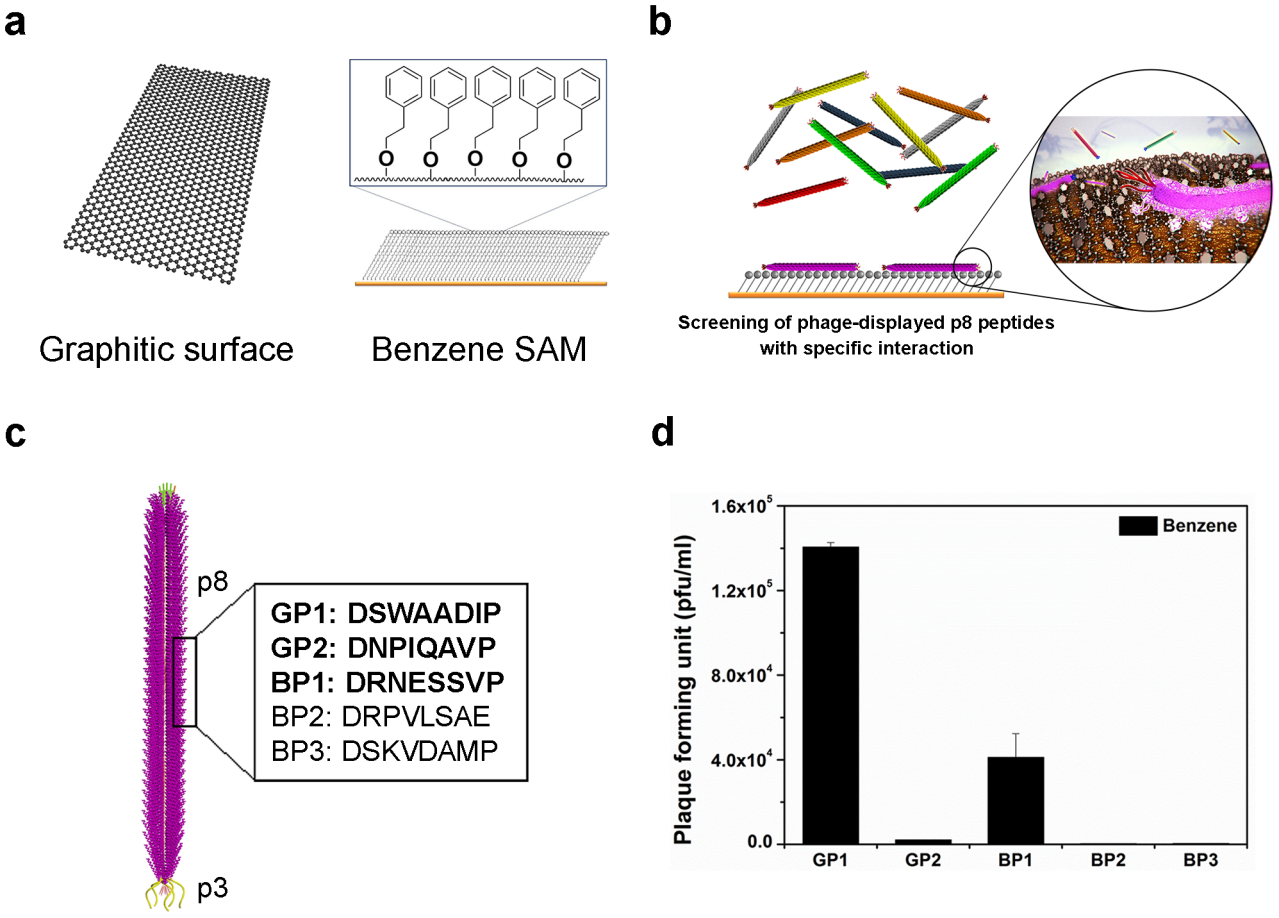
Schematic illustration of the process to screen selective peptide receptors for VOCs. (a), Schematic of the graphitic surface and the self-assembled monolayer (SAM) of phenyl-terminated alkanethiols (PTA) on an Au surface that were used as target surfaces for screening peptides toward benzene. (b), Schematic showing the selective binding of M13 phage to SAM of PTA via fusion peptide-displayed body surface (p8). (c), The amino acid sequences for the identified peptides. p3 is the minor coat protein at the tip and p8 is the major coat protein of M13 phage. GP and BP stand for graphitic surface-binding peptide and benzene-SAM binding peptide, respectively. (d), Binding test results of the identified peptides against the phenyl-functionalized SAM. The higher plaque forming unit (pfu) indicates a stronger binding affinity^26^.

Figure 1 schematically illustrates the process to screen selective peptides for VOCs. For the screening process, we utilized a phage-displayed peptide library^22^ based on the M13 phage. M13 is a filamentous phage with various capsid coat proteins that are expressed at various locations of the phage. Because of its structural simplicity and facile genetic engineering, M13 phage has been widely used for the construction of phage-displayed peptide libraries. Among various coat proteins, p3 minor coat protein, with five copies located at the tip of the phage, and p8 major coat protein, with 2,700 copies wrapping helically along the phage length, have been widely used for peptide library construction. By virtue of the huge multivalency of p8 coat proteins, a p8 peptide library is more advantageous when identifying peptides with weak binding affinity^23^. Compared with biopolymers such as proteins or oligonucleotides, small molecules such as VOCs show relatively weak affinity to their receptors. Therefore, we decided to use the phage-displayed p8 peptide-library to screen peptides against volatile small molecules. We constructed a phage-display p8 peptide library as described previously^23^,^24^ with a slight modification; BspHI and BamHI restriction enzymes were used instead of PstI and BamHI. The diversity of the constructed library was approximately 4.8 × 10^7^ plaque-forming units (PFU), and each sequence included about approximately 1.3 × 10^5^ copies (see Methods). In a general biopanning-based screening process using a phage-displayed peptide library, the library solution is incubated with a target material and phages that are bound to the target material after washing are eluted and collected. The collected phage solution gives a sub-library that is enriched for binding affinity toward the target molecules. This sub-library solution is then amplified for the next round of panning and the same process is repeated with more stringent incubating and washing conditions. After several rounds of panning, consensus sequences are identified.

In general, for the screening process the target molecules are either immobilized on a solid surface or a solid form of the target materials is used. VOCs, however, cannot be immobilized on a solid surface, nor does a solid form exist. Therefore, we prepared two different types of surface for screening against benzene as shown in Fig. 1a. In the first approach we used a highly ordered pyrolytic graphite (HOPG) surface^25^ as a relevant target surface for benzene. The surface of HOPG is composed of aromatic ring-like structures and therefore could be used as a virtual target surface for benzene. In the second approach, we used self-assembled monolayers (SAM) of phenyl-terminated alkanethiols (PTA) as a target surface for screening against benzene analogues as shown in Fig. 1b. Molecules for the SAM were synthesized (Supplementary Fig. S1), and a chip of SAM was fabricated by incubating a solution of PTA on fresh Au film for use as a target surface for the screening process (see Methods).

After several rounds of the panning process we identified several consistent peptide sequences. The results of panning are summarized in Fig. 1c. Here, GP and BP stand for graphitic surface-binding peptide and benzene-SAM binding peptide, respectively. The affinity of the identified sequences to benzene analogues was cross-checked on the SAM of PTA. Equal numbers of each selected phage expressing the peptide sequence on its body were incubated on phenyl-terminated SAM, rinsed, and eluted. The number of eluted phages was counted as PFUs; a higher PFU value indicates a stronger binding affinity toward the target surface^26^. Results of the binding test are shown in Fig. 1d. Among several identified sequences, GP1 and GP2 identified from the graphitic surface and BP1 identified from phenyl-terminated SAM were chosen on the basis of their notable binding affinity.

### Peptide receptor-immobilized microcantilever chemical sensors

**Figure 2:**
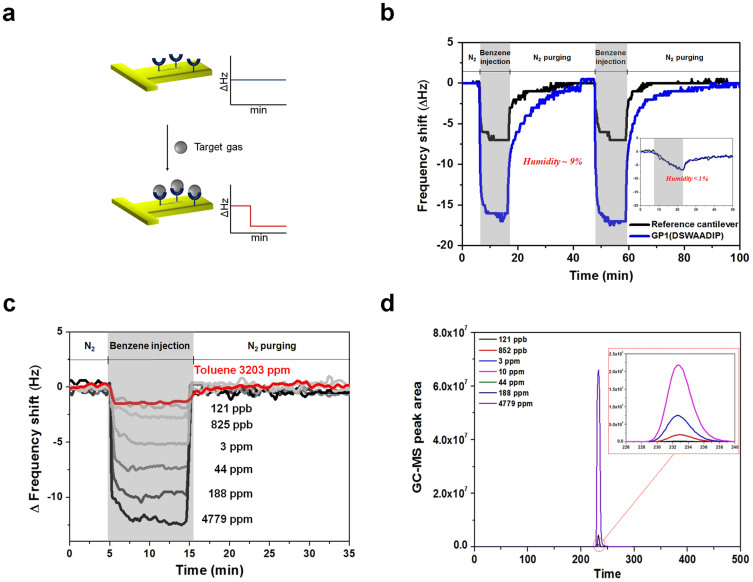
Peptide receptor-immobilized microcantilever chemical sensors. (a), Sensing scheme of the cantilever-based sensing system used in this work. The change in resonance frequency shift of peptide-immobilized cantilevers corresponds to the amount of bound target gas. (b), Representative sensing curve of the GP1 peptide-immobilized cantilever against benzene gas, compared with the curve from the reference cantilever without peptide conjugation. The humidity level was 9%. The inset shows the representative sensing curves obtained when the humidity was lower than 1%. (c), Differential frequency shift of GP1 peptide conjugated-cantilevers at various concentration levels of benzene and toluene gases. (d), Quantification of prepared benzene gas using GC-MS analysis data.

To test whether the identified peptide sequences also work for real target gases, we used the microcantilever-based sensing system reported previously^27^. For cantilever-based sensors, the recognizing molecules are immobilized on one group of cantilevers (signaling cantilevers) while the other group of cantilevers (reference cantilevers) remains intact (see Methods). When the target molecules are added to the cantilevers the resonant frequency of the signaling cantilevers downshifts more than that of the reference cantilevers as a result of the specifically bound target molecules; the differential signal between the two cantilevers is expected to correspond to the concentration of target molecules in the gas sample (Fig. 2a). In Fig. 2b, the representative sensing curve of the GP1 peptide-immobilized cantilever against benzene gas, compared with the curve from the reference cantilever without peptide conjugation is plotted as a function of time. The functionalization of the cantilevers was optimized to produce maximal selectivity and sensitivity (supplementary Fig. S2). The differential signal increased immediately after benzene injection and decreased when the injection was switched to a N_2_ supply. It should be emphasized that the kinetics of target binding and dissociation are rapid and transient, resulting in a fully recovered baseline ready for the next measurement by purging with N_2_ in only a few minutes. This reversible binding could be advantageous for continuous monitoring of VOCs in the ambient environment. As they operate at room temperature and in a repetitive manner without pre- and post-treatment, sensors with peptide receptors are suitable for a simple miniaturized system. It is noted that the peptide-immobilized signaling cantilever behaved the same way as the reference cantilever when the humidity in the gas samples was lower than 1% (inset of Fig. 2b). We suppose that, in such cases, the specific binding of benzene to peptides does not occur because water molecules are required to form the benzene-peptide complex. However, under normal ambient conditions the humidity level is greater than 10%, which is more than sufficient for target-peptide interaction. Consequently, peptides identified through biopanning can be applied for dynamic sensing of volatile molecules in ambient conditions. The differential frequency shifts of GP1 peptide conjugated-cantilevers at various concentration levels of benzene and toluene gases are presented in Fig. 2c. The prepared benzene gas was quantified using GC-MS analysis data, as shown in Fig. 2d. From the quantitative analysis, the selectivity of GP1 toward benzene over toluene is greater than 30,000-fold.

### Single-carbon discrimination by the selected peptide receptors

**Figure 3:**
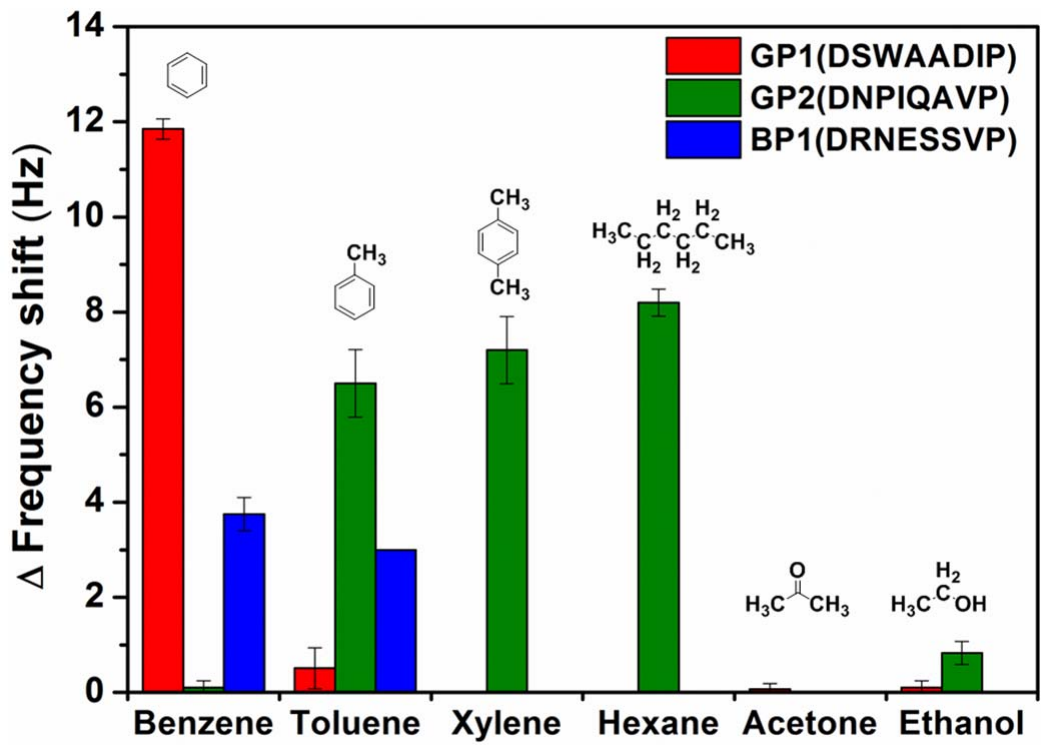
Single-carbon discrimination using selected peptides. GP1 shows extreme selectivity toward benzene over toluene, xylene, and other interfering gases. GP2 shows binding affinity toward toluene, xylene, and hexane, but not toward benzene. BP1 peptide discriminates toluene from xylene. Chemical structures for each chemical compound are shown.

To examine the selectivity of the identified peptides, several target gases such as benzene, toluene, xylene, hexane, acetone, and ethanol were applied to microcantilever-based sensors with the signaling cantilevers being fully conjugated with one of the peptides GP1, GP2 and BP1. The results summarized in Fig. 3 indicate that the peptide GP1 shows extreme selectivity toward benzene over benzene derivatives such as toluene and xylene and interfering gases such as hexane, acetone and ethanol. In contrast, the peptide GP2, which was also identified against HOPG, did not show specific binding toward benzene, but showed a very strong affinity toward toluene, xylene, and hexane. The graphitic surface of HOPG contains not only basal planes of aromatic ring-like structures, but also edges and other functional groups. Therefore, although both GP1 and GP2 were identified from biopanning against HOPG it is possible that peptide GP1 with an aromatic residue, tryptophan (W), interacted with the basal planes of HOPG via π-π interaction whereas the peptide GP2 interacted mainly with edges or functionalized spots of graphitic surfaces^28^. Therefore, our results confirmed that biopanning against solid materials with heterogeneous surfaces could be used to select heterogeneous peptide sequences that interact with various parts of the surface. In the gas sensing study with small target molecules such as benzene and toluene, the effect of this heterogeneity becomes more evident. Such extreme selectivity toward benzene over toluene has not previously been reported with peptide receptors or other approaches. This discernment between benzene and toluene might be very useful for monitoring carcinogenic benzene in the atmosphere and in screening for diseases for which benzene or toluene is used as a biomarker, such as lung cancer.

The effect of the target surface is also shown for BP1. The BP1 peptide, which was identified from the SAM surface of phenyl-terminated alkane chains, showed significant interactions with benzene and toluene, but not with xylene. This behavior could be explained in terms of the characteristics of the SAM surface. Since the carbon chain is attached to the phenyl group in the SAM, it is possible that the terminal phenyl unit replicated toluene, in which the methyl group is oriented inwards in the SAM layer. Therefore, the binding affinity of BP1 toward toluene, in addition to the intended target benzene, is presumably due to the co-existence of a toluene-like structure in PTA. However, xylene, which is characterized by two methyl substituents on the phenyl ring, might be classified differently from PTA and therefore did not bind to BP1 peptides, highlighting the fine discrimination of chemical structures by BP1. This is the first report demonstrating the capability of SAM-based surfaces for biopanning against volatile small molecules. Our data suggest that this is a general and useful approach to select receptors against small gaseous molecules when a solid form of the targeting molecules does not exist.

**Figure 4:**
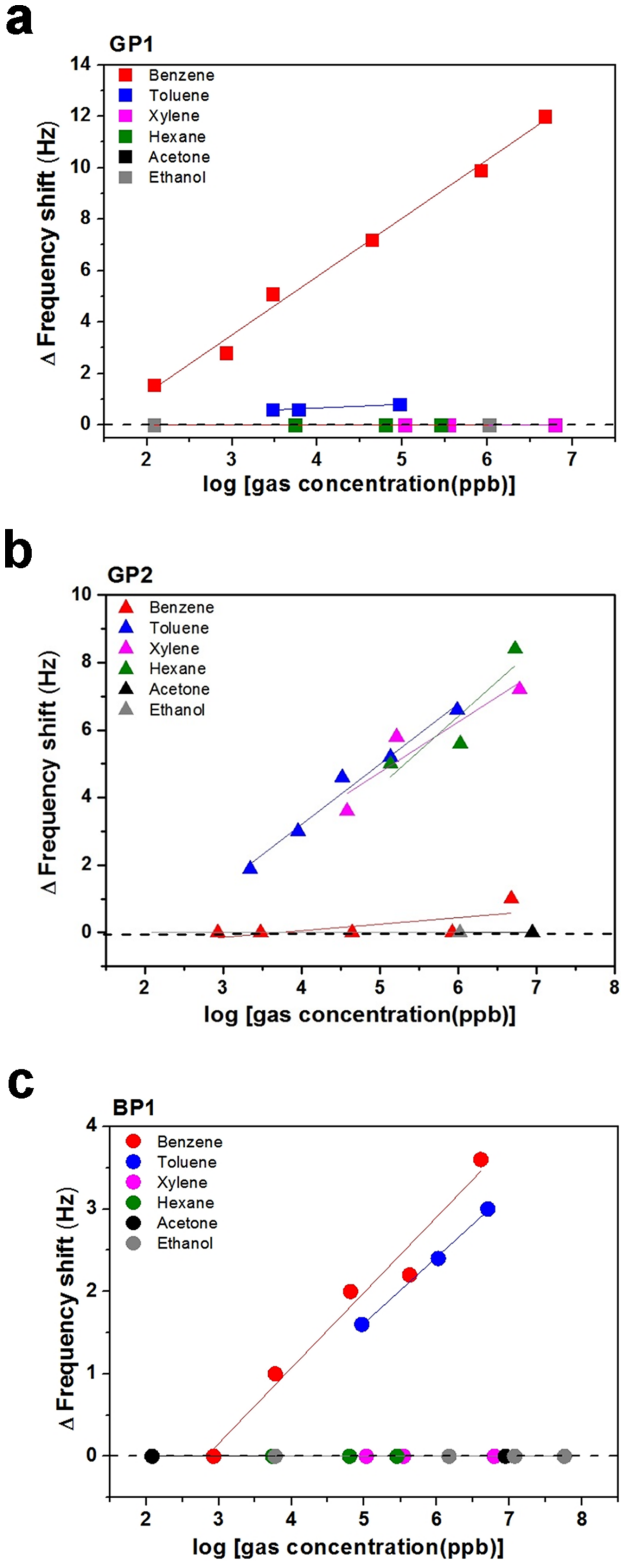
Quantitative analysis of gas sensing using selected peptides. The differential frequency shifts of peptide-conjugated microcantilevers are plotted as a function of concentration of various VOCs for GP1 (a), GP2 (b), and BP1 (c).

The sensitivity of the peptide-immobilized microcantilever sensors was further investigated. In Fig. 4, the differential frequency shift as a function of various gas concentrations in ppb units is plotted for each peptide. The concentration of each analyte was quantitated by GC-MS in parallel with measurement of the sensor. Here, it should be emphasized that the quantitative analysis shown in Fig. 4 is consistent with the selectivity results presented in Fig. 3. The GP1 peptide showed selective interaction only with benzene gas, with a response proportional to concentration down to 121 ppb in Fig. 4a. GP2-conjugated sensors could quantitate toluene, xylene and hexane to the level of 2.2 ppm, 28 ppm and 1.0 ppm, respectively. The selectivity of GP2 toward toluene over benzene was also significant, greater than 50,000-fold in Fig. 4b. The BP1 peptide showed slightly lower sensitivity than GP1 and GP2 to concentration levels of several ppm. However, BP1 showed a highly selective interaction with benzene and toluene over other gases such as xylene, hexane, acetone and ethanol in Fig. 4c. Designed selection of specific receptors for a group of small volatile molecules as demonstrated here has not been reported with other organic species^19^,^20^,^29^.

## Discussion

We have demonstrated that peptides that were selected using a p8 peptide library and phenyl-terminated SAM surfaces or graphitic surfaces successfully discriminated single-carbon deviations among benzene and its analogues such as toluene and xylene with exceptional selectivity and sensitivity. Although several studies have described peptides that bind graphitic surfaces, the potential of peptides with strong affinity toward a graphitic surface for sensing benzene analogues has never been reported. Additionally, our results using SAM surfaces suggest a general and useful strategy for the identification of receptors for sensing small gaseous molecules when a solid form of the target molecules does not exist. This study presents a systematic approach to identifying specific peptide receptors for sensing volatile small molecules and also provides feasible chemical micro-sensors with excellent selectivity and sensitivity. The exceptionally selective and sensitive interactions, as well as the different binding behaviors of selected peptide receptors could provide a very useful foundation for qualitative and quantitative sensing of various VOCs for future applications such as non-invasive testing of health conditions or environmental risk monitoring.

## Methods

### Construction of the phage-display p8 peptide library

A phage-display p8 peptide library was constructed as described previously^23^,^24^, but using BspHI and BamHI restriction enzymes instead of PstI and BamHI. A commercially available M13KE vector (New England Biolabs, product # N0316S) was subjected to site-directed mutagenesis (QuikChange Lightning Site-Directed Mutagenesis Kit, product #210518, Agilent Technologies) to change the 1381^st^ base pair, C, to G and create a BamHI recognition site, thus producing a M13HK vector. The M13HK vector was double-digested using BamHI and BspHI and dephosphorylated using Antarctic phosphatase. The dephosphorylated vector was ligated to a double-digested DNA duplex by incubation at 16°C overnight. The product was then purified and concentrated. All enzymes were purchased from New England Biolabs. Electrocompetent cells (XL-1 Blue, Stratagene) were electroporated with 2 μL of a concentrated ligated vector solution at 18 kV/cm, and a total of five transformations were performed for the library construction. The transformed cells were incubated for 60 min and fractions of several transformants were plated onto agar plates containing X-gal/isopropyl-β-D-1-thiogalactopyranoside (IPTG)/tetracycline (Tet) to determine the diversity of the library. The remaining cells were amplified for 8 h in a shaking incubator. The diversity of the constructed library was approximately 4.8 × 10^7^ plaque-forming units (PFUs), and included approximately 1.3 × 10^5^ copies of each sequence.

### Screening for benzene-binding peptides

#### Screening against HOPG surface

Peptide sequences that bound to the graphitic surfaces through the body surface peptides of M13 phage were identified by panning a phage-displayed p8 peptide library against highly ordered pyrolytic graphite (HOPG, SPI product#439HP-AB). The screening process was conducted by diluting a 4.8 × 10^10^ PFU p8 library solution in 100 μL Tris-buffered saline (TBS) containing 0.1–0.5% v/v TWEEN-20 (TBST). The library was incubated in the presence of HOPG for 30 min. The HOPG surface was washed 10 times with 1 mL TBST, and bound phages were eluted in 100 μL 0.2 M glycine-HCl (pH 2.2). After elution in a low-pH solution, the remaining phages were harvested using a mid-log *E. coli* culture and amplified for the next round of panning^30^. The number of phages used in each round was held constant and a freshly cleaved HOPG surface was used for each round. After each round, the DNA of the eluted phages was sequenced. Three rounds per biopanning experiment were performed to obtain consensus sequences. Among the identified sequences, the two strongest binders toward the graphitic surfaces, GP1 and GP2, were selected by incubating 80 μL TBST (0.1% v/v) containing 1 × 10^7^ phages, each expressing one of the sequences on the body surface, in the presence of HOPG. The number of phages that were eluted in a low-pH solution and in an *E. coli* solution was counted as PFUs. A high PFU value indicated a sequence with a high binding affinity^26^.

#### Screening against phenyl-terminated alkanethiols

Alkanethiol 7 containing a phenyl group at the terminal position was prepared in a short reaction sequence (Supplementary Fig. S1) and 1 mM phenyl-terminated alkanethiols in ethanol were immobilized on a gold surface (1 cm × 1 cm) by incubation overnight at room temperature. The modified surface was rinsed with ethanol and dried under N_2_. For the screening, 100 μL phage library solution (1 × 10^12^ PFU/mL) was treated on tri(ethylene glycol)-terminated alkanthiols (TEG) chip to remove background phage (negative selection). Supernatant phage library solution was then incubated with the phenyl-terminated alkanethiol chip at room temperature for 1 h at 50 rpm (positive selection). Unbound phages were removed by rinsing with 1 mL TBS 10 times at 100 rpm. Bound phages were eluted by incubating with 80 μL 0.2 M Glycine-HCl, pH 2.2, for 8 min, carefully transferred into 1.5-mL microcentrifuge tubes, and immediately neutralized with 20 μL 1 M Tris-HCl, pH 9.3. After buffer elution, the chip was incubated with mid-log *E.coli* solution for 30 min. The cell-eluted phages were amplified for the next round and the same procedures were repeated. Three rounds were performed per biopanning experiment to obtain consensus sequences. After each round of panning, the numbers of eluted and amplified phages, counted as PFUs, were measured using agar plates containing X-gal/IPTG/tetracycline to set the same number of input number of phage for each round. Plaques from each round were amplified and DNA was sequenced.

### Binding affinity test in liquid phase

The phenyl-terminated alkanethiols chip was incubated with 100 μL phage (1 × 10^9^ PFU) for 1 h. The benzene surface was rinsed with 1 mL TBS five times at 50 rpm to remove unbound phages. Bound phages were eluted by incubation with 80 μL 0.2 M Glycine-HCl, pH 2.2, for 8 min, carefully transferred into 1.5-mL centrifuge tubes, and immediately neutralized with 20 μL 1 M Tris-HCl, pH 9.3. The eluted phage-solutions were serially diluted and plated on agar plates containing X-gal/IPTG/tetracycline. The plates were incubated at 37°C overnight and the numbers of eluted phages were counted as PFU from blue plaques.

### Gas sensing using a microcantilever system

A microcantilever system was used for verification of the molecular interaction between benzene analogues and the specific screened peptides. The microcantilever system was prepared as described previously^27^. Briefly, the cantilever system consists of four compartments, with each compartment including three cantilevers. Separate compartment enables independent functionalization of the cantilevers. Cantilevers in the fourth compartment were used as references. For peptide immobilization, Cr (10 nm)/Au (50 nm) layers were deposited onto the microcantilevers. The surface was cleaned in piranha solution (4:1 ratio of H_2_SO_4_ (98.08%) and H_2_O_2_ (34.01%)) to remove any contaminants present on the surface, and then rinsed with deionized (DI) water. Thiolated peptides (50 μL of 10 μM solution) were immobilized on the gold surface of cantilevers at room temperature for 5 h. The peptide-conjugated microcantilevers were rinsed with DI/ethanol and dried under N_2_. For measurements, the peptide-conjugated microcantilevers were enclosed within a chamber containing an inlet and an outlet for the gas flow. Humidity during measurements was monitored with an integrated sensor in the chamber. The temperature was controlled at 20°C with a thermoelectric cooler that was also integrated to the chamber. We precisely controlled the flow rate of all gases at 100 standard cubic centimeter per minute (sccm) using a mass flow controller (MFC). Before the measurement, the microcantilevers were stabilized by N_2_ at 100 sccm overnight. Target gas was blown into the measurement chamber for 10 min. After target injection, the microcantilever in the chamber was purged with N_2_ for 25 min and the next target gas was introduced into the chamber sequentially.

### Quantitative analysis of gases using GC-MS

Dry gases, with humidity less than 1%, were directly injected from the standard gases (10 ppm, >95%). Except the dry samples, stock samples of individual target gases were generated by injecting N_2_ into each bottle containing the target molecule in liquid phase. Each stock gas was collected in a Tedlar bag (5 L; Top Trading Eng Co., Ltd. Seoul, Korea). Target samples of different concentrations were prepared by serially diluting the stock sample with N_2_ to the designated concentration. For quantitative calibration of the target samples, each sample was analyzed by gas chromatography–mass spectrometry (GC: 6890N GC system, Agilent Tech, Santa Clara, USA) that was calibrated with 99.5% grade standard gases. GC-MS analytical parameters are as follows: temperature: 260°C, volume: 20 μL, carrier gas flow rate: 0.8 mL/min, column: ZB-5ms (30 × 0.25 × 0.25) (Zebron, Phenomenex, USA).

## Author Contributions

S.J., K.Y.L., S.K.K. and H.Y. conceived the idea and, together with S.J.M. and K.S.H. designed the experiments. S.J., K.Y.L. and Y.K.Y. performed the experiments. S.J., S.K.K. and H.Y. co-wrote the manuscript and all authors discussed the results and commented on the manuscript.

## Supplementary Material

Supplementary Information Supplementary Information

## References

[b1] Smith M. T. The mechanism of benzene-induced leukemia: a hypothesis and speculations on the causes of leukemia. Environmental health perspectives 104 **Suppl 6**, 1219–1225 (1996). 9118896 10.1289/ehp.961041219 PMC1469721

[b2] Snyder R. Benzene and leukemia. Critical reviews in toxicology 32, 155–210 (2002). 12071572 10.1080/20024091064219

[b3] van de Kant K. D. G., van der Sande L. J. T. M., Jobsis Q., van Schayck O. C. P. & Dompeling E. Clinical use of exhaled volatile organic compounds in pulmonary diseases: a systematic review. Resp Res 13, 117–139 (2012). 10.1186/1465-9921-13-117 PMC3549749 23259710

[b4] Zhou M., Liu Y. & Duan Y. Breath biomarkers in diagnosis of pulmonary diseases. Clinica chimica acta; international journal of clinical chemistry 413, 1770–1780 (2012). 10.1016/j.cca.2012.07.006 22796631

[b5] Buszewski B., Kesy M., Ligor T. & Amann A. Human exhaled air analytics: biomarkers of diseases. Biomedical chromatography: BMC 21, 553–566 (2007). 17431933 10.1002/bmc.835

[b6] Van Berkel J. J. *et al.* A profile of volatile organic compounds in breath discriminates COPD patients from controls. Respiratory medicine 104, 557–563 (2010). 19906520 10.1016/j.rmed.2009.10.018

[b7] Moon H. G. *et al.* Self-activated ultrahigh chemosensitivity of oxide thin film nanostructures for transparent sensors. Sci. Rep. 2, 588–594 (2012). 22905319 10.1038/srep00588 PMC3421433

[b8] Maier K. *et al.* Detection of oxidising gases using an optochemical sensor system based on GaN/InGaN nanowires. Sensor Actuat B-Chem 197, 87–94 (2014).

[b9] Patil J. Y., Nadargi D. Y., Gurav J. L., Mulla I. S. & Suryavanshi S. S. Glycine combusted ZnFe2O4 gas sensor: Evaluation of structural, morphological and gas response properties. Ceram Int 40, 10607–10613 (2014).

[b10] Kim H. J. *et al.* Ultraselective and sensitive detection of xylene and toluene for monitoring indoor air pollution using Cr-doped NiO hierarchical nanostructures. Nanoscale 5, 7066–7073 (2013). 23807747 10.1039/c3nr01281f

[b11] Zhang F. H., Wang X. H., Dong J. P., Qin N. & Xu J. Q. Selective BTEX sensor based on a SnO2/V2O5 composite. Sensor Actuat B-Chem 186, 126–131 (2013).

[b12] Lin H. W., Jang M. & Suslick K. S. Preoxidation for Colorimetric Sensor Array Detection of VOCs. Journal of the American Chemical Society 133, 16786–16789 (2011). 21967478 10.1021/ja207718t PMC3197745

[b13] Oo M. K. K., Guo Y. B., Reddy K., Liu J. & Fan X. D. Ultrasensitive Vapor Detection with Surface-Enhanced Raman Scattering-Active Gold Nanoparticle Immobilized Flow-Through Multihole Capillaries. Anal Chem 84, 3376–3381 (2012). 22413933 10.1021/ac300175v

[b14] Wang J., Yang L. L., Boriskina S., Yan B. & Reinhard B. M. Spectroscopic Ultra-Trace Detection of Nitroaromatic Gas Vapor on Rationally Designed Two-Dimensional Nanoparticle Cluster Arrays. Anal Chem 83, 2243–2249 (2011). 21332229 10.1021/ac103123r

[b15] Lee D., Kim S., Jeon S. & Thundat T. Direct Detection and Speciation of Trace Explosives Using a Nanoporous Multifunctional Microcantilever. Anal Chem 86, 5077–5082 (2014). 24766474 10.1021/ac500745g

[b16] Peng G., Trock E. & Haick H. Detecting Simulated Patterns of Lung Cancer Biomarkers by Random Network of Single-Walled Carbon Nanotubes Coated with Nonpolymeric Organic Materials. Nano Lett 8, 3631–3635 (2008). 18839997 10.1021/nl801577u

[b17] Raorane D., Lim S. H. S. & Majumdar A. Nanomechanical assay to investigate the selectivity of binding interactions between volatile benzene derivatives. Nano Lett 8, 2229–2235 (2008). 18616329 10.1021/nl080829s

[b18] Kybert N. J., Lerner M. B., Yodh J. S., Preti G. & Johnson A. T. C. Differentiation of Complex Vapor Mixtures Using Versatile DNA-Carbon Nanotube Chemical Sensor Arrays. Acs Nano 7, 2800–2807 (2013). 23442175 10.1021/nn400359c

[b19] Mirica K. A., Azzarelli J. M., Weis J. G., Schnorr J. M. & Swager T. M. Rapid prototyping of carbon-based chemiresistive gas sensors on paper. P Natl Acad Sci USA 110, E3265–E3270 (2013). 10.1073/pnas.1307251110 PMC3761578 23942132

[b20] Kim T. H. *et al.* Single-Carbon-Atomic-Resolution Detection of Odorant Molecules using a Human Olfactory Receptor-based Bioelectronic Nose. Adv Mater 21, 91–94 (2009).

[b21] Jaworski J. W., Raorane D., Huh J. H., Majumdar A. & Lee S. W. Evolutionary screening of biomimetic coatings for selective detection of explosives. Langmuir 24, 4938–4943 (2008). 18363413 10.1021/la7035289

[b22] Barbas C. F., Burton D. R., Scott J. K. & Silverman G. J. Phage Display: A Laboratory Manual. (CSH Press, 2001).

[b23] Petrenko V. A., Smith G. P., Gong X. & Quinn T. A library of organic landscapes on filamentous phage. Protein Eng 9, 797–801 (1996). 8888146 10.1093/protein/9.9.797

[b24] Lee S. K., Yun D. S. & Belcher A. M. Cobalt ion mediated self-assembly of genetically engineered bacteriophage for biomimetic Co-Pt hybrid material. Biomacromolecules 7, 14–17 (2006). 16398491 10.1021/bm050691x

[b25] So C. R. *et al.* Controlling Self-Assembly of Engineered Peptides on Graphite by Rational Mutation. Acs Nano 6, 1648–1656 (2012). 22233341 10.1021/nn204631x PMC3304023

[b26] Lee Y. J. *et al.* Fabricating Genetically Engineered High-Power Lithium-Ion Batteries Using Multiple Virus Genes. Science 324, 1051–1055 (2009). 19342549 10.1126/science.1171541

[b27] Hwang K. S. *et al.* Peptide receptor-based selective dinitrotoluene detection using a microcantilever sensor. Biosens Bioelectron 30, 249–254 (2011). 22000759 10.1016/j.bios.2011.09.021

[b28] Kim S. N. *et al.* Preferential Binding of Peptides to Graphene Edges and Planes. J Am Chem Soc 133, 14480–14483 (2011). 21861527 10.1021/ja2042832

[b29] Ablat H., Yimit A., Mahmut M. & Itoh K. Nafion film/K(+)-exchanged glass optical waveguide sensor for BTX detection. Anal Chem 80, 7678–7683 (2008). 18781774 10.1021/ac800815g

[b30] Dang X. N. *et al.* Virus-templated self-assembled single-walled carbon nanotubes for highly efficient electron collection in photovoltaic devices. Nat Nanotechnol 6, 377–384 (2011). 21516089 10.1038/nnano.2011.50

