# Supplementary material for: Single-carbon discrimination by selected peptides for individual detection of volatile organic compounds
Source: Sci Rep. 2015 Mar 17;5:9196. doi: 10.1038/srep09196 (PMC4361882; doi:10.1038/srep09196)
Supplement: Supplementary Information [file srep09196-s1.pdf]

Supplementary Information for

Single-carbon discrimination by selected peptides for individual detection of volatile organic compounds

Soomi Ju[1+], Ki-Young Lee[2+], Sun-Joon Min[3], Yong Kyoung Yoo[1], Kyo Seon Hwang[1]*, Sang Kyung Kim[1]* and Hyunjung Yi[2]*

[1]Center for BioMicrosystems, Brain Science Institute, Korea Institute of Science and Technology, Seoul, 136-791, Republic of Korea.

[2]Center for Spintronics Research, Post-Silicon Semiconductor Institute, Korea Institute of Science and Technology, Seoul, 136-791, Republic of Korea

[3]Center for Neuro-medicine, Brain Science Institute, Korea Institute of Science and Technology, Seoul, 136-791, Republic of Korea.

[+] These authors contributed equally.

* kshwang@kist.re.kr, sangk@kist.re.kr, hjungyi@kist.re.kr,

Includes:

1) Two supplementary figures

2) Supplementary methods

3) Supplementary references

Supplementary Figures

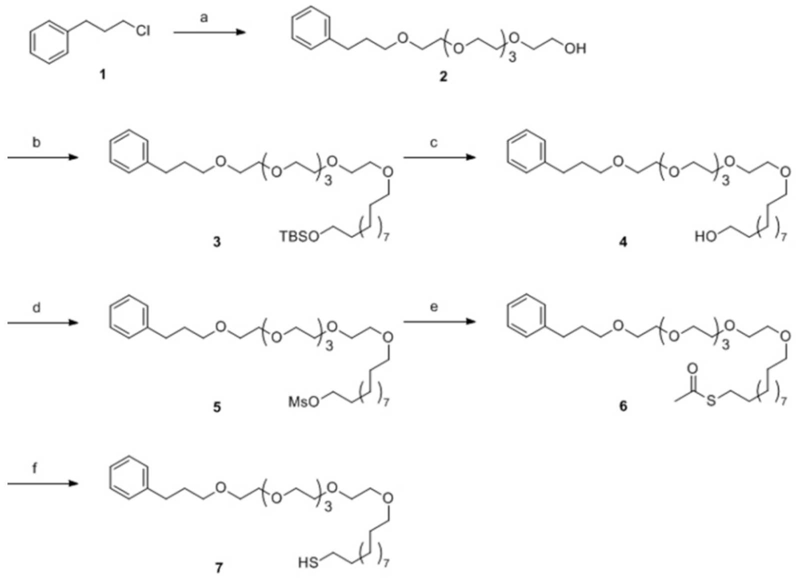

Figure S1. Synthesis of phenyl terminated alkanethiol (PTA): (7): a) pentaethylene glycol, NaH, KI, DMF, 0 °C to rt, 65%; b) TBSO(CH2)11Br, NaH, DMF, 0 °C to rt , 47%; c) TBAF, THF, rt, 97%; d) MsCl, Et3N, CH2Cl2, rt, 92%; e) CH3COSK, DMF, rt, 79%; f) HCl, MeOH, 60 °C, 96%. See Supplementary Methods.

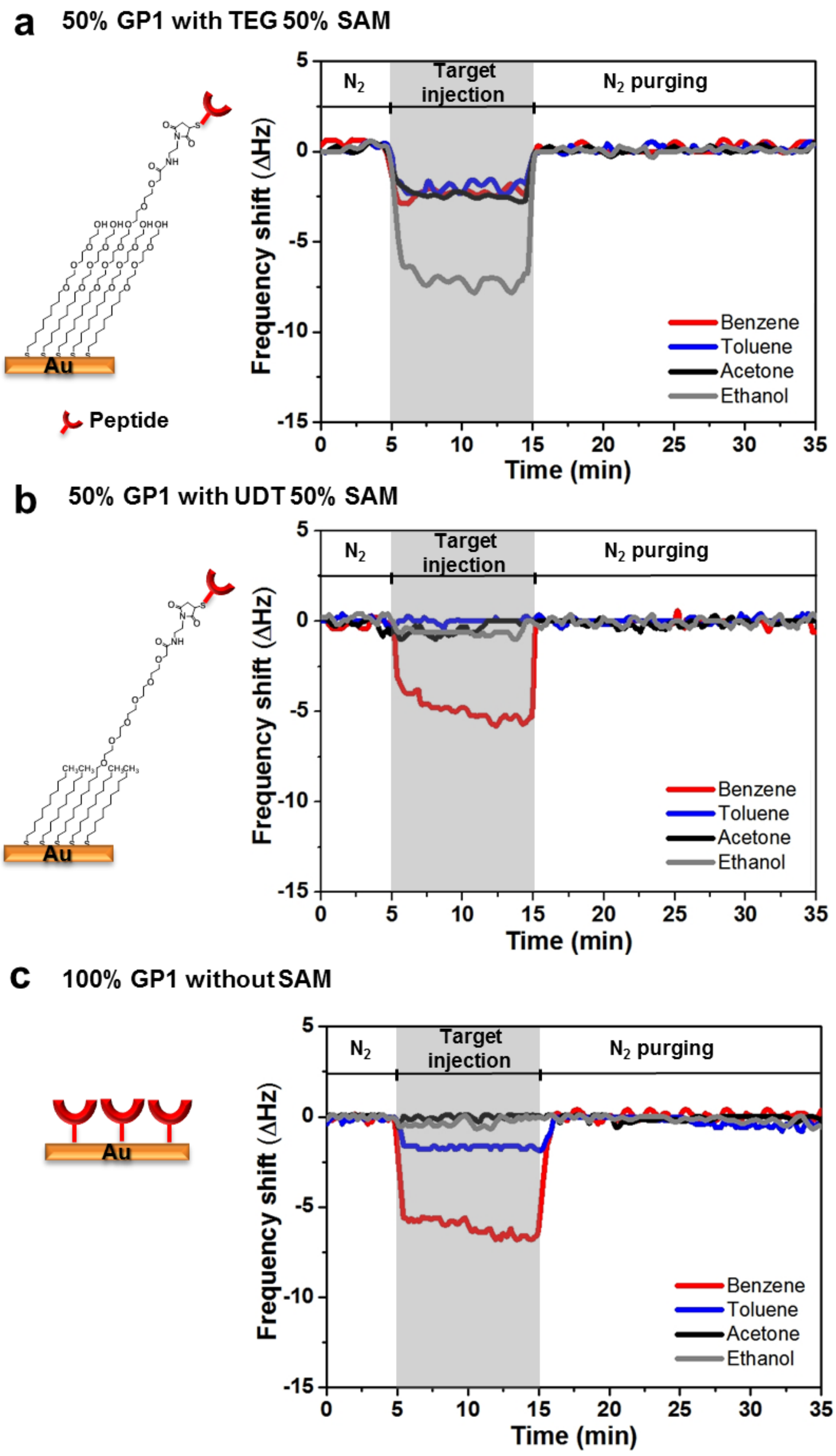

Figure S2. Frequency shift of GP1 peptide-conjugated microcantilever with 50% TEG SAM (a), 50% UDT SAM (b), and without back-filler (c).

Supplementary Methods

Construction of the phage-display p8 peptide library

A phage-display p8 peptide library was constructed as described previously[1,2] with slight modification. The BspHI and BamHI restriction enzymes were used in place of PstI and BamHI. A commercially available M13KE vector (New England Biolabs. NEB, product # N0316S) was site-directed mutated (QuikChange Lightning Site-Directed Mutagenesis Kit, product #210518, Agilent Technologies) to change the 1381st base pair C to G to create BamH I recognition site, producing a M13HK vector. The nucleotide sequences used for the site- mutagenesis were 5'-AAG GCC GCT TTT GCG GGA TCC TCA CCC TCA GCA GCG AAA GA-3' and 5'-TCT TTC GCT GCT GAG GGT GAG GAT CCC GCA AAA GCG GCC TT-3'. The M13HK vector was double-digested using BamHI and BspHI and then dephosphorylated using Antarctic phosphatase. The dephosphorylated vector was ligated to a double cut- DNA duplex with incubation at 16℃ overnight. The product was then purified and concentrated. (All enzymes were purchased from New England Biolabs.). The oligonucleotide sequences used to create the DNA-duplex inserts were 5'-TTA ATG GAA ACT TCC TCA TGA AAA AGT CTT TAG TCC TCA AAG CCT CTG TAG CCG TTG CTA CCC TCG TTC CGA TGC TGT CTT TCG CTG CTG -3' and 5'-AAG GCC GCT TTT GCG GGA TCC NNM NNM NNM NNM NNM NNM NNM NCA GCA GCG AAA GAC AGC ATC GGA ACG AGG GTA GCA ACG GCT ACA GAG GCT TT -3'. Two 2 μL of a concentrated ligated vector solution was electro-transformed into electro-competent cells (XL-1 Blue, Stratagene) at 18 kV/cm, and a total of five transformations were performed for the library construction. The transformed cells were incubated for 60 min, and fractions of several transformants were plated onto agar plates containing X-gal/isopropyl-β-D-1-thiogalactopyranoside (IPTG)/tetracycline (Tet) in order to count the diversity of the library. The remaining of the cells was amplified for 8 h in a shaking incubator. The diversity of the constructed library was about 4.8×10^7 pfu, and each sequence included about 1.3×10^5 copies.

Synthesis of phenyl-terminated alkanethiol (PTA)

As shown in Figure Sx, alkanethiol 7 containing a phenyl group at the terminal position was prepared in a short reaction sequence. Initially, treatment of (3-chloropropyl)benzene 1 with pentaethylene glycol produced the primary alcohol 2,

which was subjected to etherification with 11-bromo-1-undecyl tert-butyldimethylsilyl ether[3] to give silyl ether 3. Removal of the TBS protecting group of 3 with tetrabutylammonium fluoride provided alcohol 4, which was subsequently transformed to the corresponding mesylate 5 in high yield. Finally, we obtained the desired alkanethiol 7 by a displacement reaction of 5 with potassium thioacetate followed by acid-promoted hydrolysis.

Optimization of functionalization of cantilevers with various compositions of peptides and back-fillers

1) For the surface functionalization with 50% peptide and 50% TEG: Au layer-coated cantilevers were incubated with a mixed solution of tri(ethylene glycol)-terminated alkanethiol (TEG) (1 mM in ethanol) and acid penta(ethylene glycol)-terminated alkanethiol (1 mM in ethanol) in a ratio of 1:1 for 12 h. The resulting cantilevers were washed with absolute ethanol. The acid-presenting cantilevers with a density of 50% were treated with 40 μl of N-aminoethyl maleimide (7 mg/ml in PBS) and 40 μl of EDC (20 mg/ml in PBS) for 1 h and rinsed with DI water and ethanol sequentially and dried under N_2 blow. Thiolated peptides (50 μl of 10 μM in PBS) were immobilized on the cantilevers at room temperature for 5 h and washed with DI/ethanol and dried under N_2.

2) For the surface functionalization with 50% peptide and 50% UDT: Au layer-coated cantilevers were incubated with a mixed solution of 1-undecanethiol (UDT) (1 mM in ethanol) and acid penta(ethylene glycol)-terminated alkanethiol (1 mM in ethanol) in a ratio of 1:1 for 12 h. The subsequent process of peptide binding to self-assembled monolayer was the same as in the case of 50% peptide and 50% TEG.

3) For the surface functionalization with 100% peptide, thiolated peptides (50 μl of 10 μM peptides in PBS) were immobilized on the gold surface of cantilevers at room temperature for 5 h. The peptide-conjugated microcantilevers were rinsed with DI water and ethanol sequentially and dried under N_2 blow.

Supplementary References

1 Petrenko, V. A., Smith, G. P., Gong, X. & Quinn, T. A library of organic landscapes on filamentous phage. *Protein Eng* **9**, 797-801 (1996).

2 Lee, S. K., Yun, D. S. & Belcher, A. M. Cobalt ion mediated self-assembly of genetically engineered bacteriophage for biomimetic Co-Pt hybrid material. *Biomacromolecules* **7**, 14-17 (2006).

3 Barriet, D., Chinwangso, P. & Lee, T. R. Can Cyclopropyl-Terminated Self-Assembled Monolayers on Gold Be Used to Mimic the Surface of Polyethylene? *Acs Appl Mater Inter* **2**, 1254-1265, (2010).
